# Stress recovery of laminated non-prismatic beams under layerwise traction and body forces

**DOI:** 10.1007/s10999-022-09601-0

**Published:** 2022-07-18

**Authors:** M. M. S. Vilar, D. A. Hadjiloizi, P. Khaneh Masjedi, P. M. Weaver

**Affiliations:** 1 Bernal Institute, School of Engineering, University of Limerick, Limerick, Ireland; 2 Department of Aerospace and Mechanical Engineering, South East Technological University, Carlow Campus, Carlow, Ireland

**Keywords:** Non-prismatic beam, Tapered beam, Laminated beam, Layerwise load, Traction, Stress recovery

## Abstract

Emerging manufacturing technologies, including 3D printing and additive layer manufacturing, offer scope for making slender heterogeneous structures with complex geometry. Modern applications include tapered sandwich beams employed in the aeronautical industry, wind turbine blades and concrete beams used in construction. It is noteworthy that state-of-the-art closed form solutions for stresses are often excessively simple to be representative of real laminated tapered beams. For example, centroidal variation with respect to the neutral axis is neglected, and the transverse direct stress component is disregarded. Also, non-classical terms arise due to interactions between stiffness and external load distributions. Another drawback is that the external load is assumed to react uniformly through the cross-section in classical beam formulations, which is an inaccurate assumption for slender structures loaded on only a sub-section of the entire cross-section. To address these limitations, a simple and efficient yet accurate analytical stress recovery method is presented for laminated non-prismatic beams with arbitrary cross-sectional shapes under layerwise body forces and traction loads. Moreover, closed-form solutions are deduced for rectangular cross-sections. The proposed method invokes Cauchy stress equilibrium followed by implementing appropriate interfacial boundary conditions. The main novelties comprise the 2D transverse stress field recovery considering centroidal variation with respect to the neutral axis, application of layerwise external loads, and consideration of effects where stiffness and external load distributions differ. A state of plane stress under small linear-elastic strains is assumed, for cases where beam thickness taper is restricted to $15^{\circ }$. The model is validated by comparison with finite element analysis and relevant analytical formulations.

## Introduction

Laminated non-prismatic, or multilayered tapered, beams are variable cross-section slender structures comprising multiple materials stacked in layers in a specific order (Balduzzi et al. 2017a, 2016). To manufacture tapered beams, modern technologies such as 3D printing and automated welding can shape beam-like structures of complex geometry. As a result, more cost-efficient structural elements can be produced by optimizing stiffness distributions (Timoshenko and Young 1945; Bai et al. 2018; Vo et al. 2021) while meeting design criteria. Regarding the manufacture of tapered composite laminates, two methods are employed: ply drop-off and variable pre-preg tape spreading. The ply drop-off technique has gained attention during the late twentieth century, and involves terminating constant thickness plies with associated resin pocket tapering (Curry et al. 1992; Mukherjee and Varughese 2001; Varughese and Mukherjee 1997; Sudhagar et al. 2017). However, eliminating layers results in stress concentrations due to cutting of load bearing fibers (Varughese and Mukherjee 1997; Her 2002). Conversely, the variable tape spreading technique developed by Clancy et al. (2020) gradually introduces tapering to laminates composed of carbon fiber pre-impregnated with thermoplastic resin. This technique utilizes laser-assisted tape placement (LATP) in conjunction with a spreading device before consolidation takes place, thus avoiding stress concentrations by doing so. As technologies emerge to manufacture heterogeneous tapered beams, sophisticated analytical methods are required to comprehend the mechanical behavior of innovative structures. Hence this investigation focuses on laminated non-prismatic beams with variable thickness layers.

In addition to being lighter, non-prismatic beams can be manufactured to meet specific geometrical shapes driven by design requirements. Well-known examples include bridges, helicopter and wind turbine blades and aircraft wings (Hodges 1990; Balduzzi et al. 2017d; Vinod et al. 2007; Vo et al. 2021). Nevertheless, predicting tapered beam behavior through 1D beam formulations poses some challenges. For example, the state of stress on beam surfaces is governed by Cauchy’s traction-stress relation, resulting in non-vanishing transverse stresses (Balduzzi et al. 2016, 2017a, b, c, d, 2019; Mercuri et al. 2020; Hodges et al. 2008, 2011; Vilar et al. 2021a, b). For heterogeneous media, Cauchy’s traction-stress equation must also be satisfied on interface surfaces, which causes transverse stress discontinuities (Balduzzi et al. 2017a). Furthermore, beam eccentricity, which describes the offset of centroidal position from the neutral axis, plays an essential role in deformation and stress distribution (Balduzzi et al. 2016), but is often neglected in non-prismatic beam formulations. Another drawback is that the Euler–Bernoulli beam theory assumes the external load is reacted uniformly through the cross-section, which is referred to in this work as generalizing the external load. This hypothesis, however, is not appropriate to cases where the external load is applied to a portion of the cross-sectional area. Practical examples include aircraft wings, wind turbine and helicopter rotor blades under fluid pressure and shear forces; arched beams in bridges under vehicular traction forces and tensile stresses in tendons of prestressed concrete (Vilar et al. 2021b). As such, an accurate and effective analytical method for stresses is necessary to exploit the full potential of laminated non-prismatic beams.

Various analytical solutions have been proposed to predict the stress field of homogeneous non-prismatic beams. Bleich (1932) derived the shear stress of linearly tapered beams based on a generalization of the parabolic shear stress distribution of prismatic beams, also known as Jourawski’s formula (Jourawski 1856). Subsequently, many investigations proposed shear stress recovery procedures to infinite wedge elements via Theory of Elasticity approaches (Michell 1900; Carothers 1914; Timoshenko and Goodier 1951), and later to truncated linearly tapered beams (Krahula 1975), including that of I-sections (Blodgett 1966; Vu-Quoc and Léger 1992; Trahair and Ansourian 2016; Balduzzi et al. 2017b). Boley (1963), using Airy’s stress function, examined the limits of a linear longitudinal stress distribution in non-prismatic beams under pure bending, and concluded that the error associated with Navier’s hypothesis escalates with the taper angle, such that a $10^{\circ }$-taper results in $7.5\%$ of error in predicting the longitudinal stress. Romano (1996) offered a solution to shear stress for linearly tapered beams using Timoshenko beam theory. Hodges et al. (2008, 2011) used the variational asymptotic method to model linearly tapered beams under extension, bending, and transverse loads. Furthermore, Beltempo et al. (2015) employed an energy-based approach in conjunction with the Hellinger-Reissner Principle to predict the mechanical behavior of non-prismatic beams. Later, Mercuri et al. (2020) used the Hellinger-Reissner Principle to propose solutions for the full 2D stress field. Balduzzi et al. (2016) reconstructed the shear stress of tapered beams by superposing Jourawski’s formula to a linear function that satisfies traction-free boundary equilibrium on oblique surfaces. (Zhou et al. 2016, 2020b) predicted the shear stress of asymmetric linearly tapered beams with box girder and rectangular cross-sections, respectively. Moreover, Taglialegne (2018) and Bertolini et al. (2019) derived the stress field of symmetric linearly tapered thin-walled beams. Subsequently, Bertolini and Taglialegne (2020) proposed a similar methodology to include taper in the width direction. Chockalingam et al. (2020) derived the shear stress of I-beams tapered in the thickness and width directions. Also, Vilar et al. (2021a, 2021b) recovered the 2D stress field of non-prismatic beams subject to generalized and partial cross-sectional loads, respectively.

In contrast to homogeneous media, the fewer investigations concerning laminated tapered beams leave scope for important limitations to be addressed. For example, Balduzzi et al. (2017a) proposed a solution for shear stress and displacements in laminated tapered beams under plane stress. However, beam eccentricity and the spanwise distributed bending moment arising from eccentric axial loading are disregarded in the stress recovery procedure, although both are included in the equilibrium relations of generalized forces and moments. Ai and Weaver (2017) modeled tapered sandwich beams with a functionally graded core based on a layerwise displacement field approximated via the Ritz method, yet the beam shape is limited to being symmetric with respect to the longitudinal axis. Zhou et al. (2020a, 2021) proposed solutions for shear stress in non-prismatic beams with a corrugated steel web considering the Resal effect, i.e., the reduction of the effective shear force due to compressive forces acting on the tapered flange, and shear lag analysis, respectively. However, in both investigations, cross-sections were restricted to thin-walled members. In general, classical beam models generalize the external load through the entire cross-section. This hypothesis, although not best representing surface forces and partial cross-sectional loads, is widely accepted in beam modeling approaches (Masjedi et al. 2019; Masjedi and Weaver 2020a, b, 2022; Masjedi et al. 2021; Doeva et al. 2020a, b, 2021, 2022; Balduzzi et al. 2019; Bertolini et al. 2019; Bertolini and Taglialegne 2020; Chockalingam et al. 2020, 2021; Vilar et al. 2021a). Another issue not addressed in analytical laminated beam formulations is for cases where the stiffness distribution is different from the transverse and axial load distributions, which gives rise to non-classical transverse stresses. More specifically, derivatives of the axial force, i.e., the generalized longitudinal stress, are required to predict the transverse stresses: the first derivative for the shear component and up to the second for the transverse direct stress. Additionally, the first derivative of the shear force is necessary to define the transverse direct stress, noting that the contribution of this term differs from that of prismatic beams.

To remedy the state-of-the-art limitations, we provide an analytical stress recovery procedure to laminated non-prismatic beams subject to layerwise body forces and traction loads. Beam eccentricity is accounted for in the equilibrium equations and stress-field derivation. Non-classical terms arise from cross-sectional interactions between stiffness distribution, and the transverse and longitudinal external load distributions. The cross-sectional shape is arbitrary but has simplifications, including cross-sections with no cut-outs and interfacial surface projections are parallel to the global reference system. To give insight into the potential of the developed formulation, closed-form solutions are deduced for the case of a rectangular cross-section.

The proposed method can be applied to untwisted laminated beams tapered in the thickness direction with perfectly bonded interfaces. A state of plane stress is assumed, coupled with linear-elastic material undergoing small strains. A linear longitudinal stress distribution with discontinuities at interfaces is adopted. Subsequently, the transverse stress field is recovered following Jourawski’s formulation adapted to heterogeneous non-prismatic beams. In the context of analytical methods for laminated non-prismatic beams, the main novelties of the proposed approach include: (1) the recovery of the shear stress distribution considering beam eccentricity; (2) consideration of non-classical terms originating from the mismatch between stiffness and external load cross-sectional distributions; (3) derivation of the transverse direct stress component and (4) application of layerwise loading and traction forces.

The outline of this work is as follows: Sect. 2 provides the hypotheses and limitations as well as the equilibrium relations. Section 3 recovers the transverse stress field. Section 4 compares results of the developed theory with relevant analytical formulations and finite element analyses. Finally, Sect. 5 summarizes the novelties of this study and suggests further developments.

## Problem idealization

This section introduces the underlying principles of the proposed theory. Herein the Cartesian coordinate system is adopted such that the *x*-axis is parallel to the beam axis and the *y*- and *z*- axes are parallel to the beam width and height (or thickness), respectively.

**Fig. 1:**
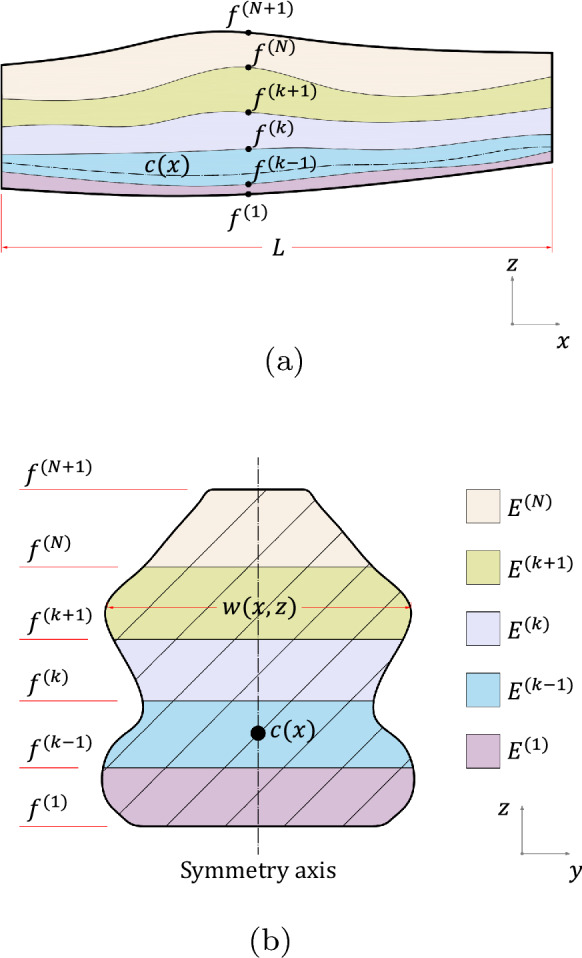
**a** Side perspective. **b** Generic cross-section

The object of study is a laminated non-prismatic beam comprising *n* layers as depicted in Fig. 1. The *xz*-plane is under plane stress undergoing small displacements and linear strains. Also, the cross-section of the beam remains plane after deformation but not necessarily perpendicular to the neutral axis, thus consistent with the First Order Shear Deformation Theory. It is assumed that the beam length *L* is significantly greater than the depth *h*(*x*) and width *w*(*x*, *z*) to meet the slenderness requirements of beam theory.

To be consistent with the hypotheses assumed, the following limitations are imposed to the cross-sectional shape:

1. symmetry with respect to the *z*-axis is adopted to avoid cross-section warping effects;
2. the ratio *w*/*h* should be sufficiently small to reduce shear stress in the *yz*-plane (Timoshenko and Goodier 1951), noting that an optimized ratio depends on specific cross-sectional shapes;
3. $\partial w/\partial x\approx 0$ to be consistent with the plane stress hypothesis;
4. interfacial boundaries are perfectly bonded and their projections to the cross-section are parallel to the *xy*-plane;
5. cut-outs are disregarded to avoid stress concentrations.

Introducing $(n+1)$ *interface surfaces* $f^{(k)}$, that represent the *z*-coordinate of the *k*th layer’s lower interface, note that $f^{(n+1)}$ and $f^{(1)}$ correspond to the upper and lower beam surfaces, respectively (dependency on the *x*-coordinate is omitted). The taper angle, i.e., the angle formed by the *x*-axis and the plane tangent to the beam surfaces, is smaller than $15^{\circ }$ throughout the longitudinal axis for reasons explained in Sect. 3.

The adopted geometrical description implies that cross-sections are non-orthogonal to the beam’s neutral axis. Instead, orthogonality is preserved in the Cartesian coordinate system, noting similar methodology is adopted in related works (Balduzzi et al. 2016; Gimena et al. 2008; Rajagopal and Hodges 2014; Balduzzi et al. 2017a, c, d; Mercuri et al. 2020; Vilar et al. 2021a). Consequently, the cross-sectional layer area $A^{(k)}$ and total cross-sectional area *A* are given by

2.1 $$\begin{aligned} \begin{aligned}&A^{(k)}(x)= \int _{f^{(k)}}^{f^{(k+1)}}\int _{w(x,z)}\mathrm {d}y\mathrm {d}z\\&A(x) = \sum _{k=1}^{n}A^{(k)} \end{aligned} \end{aligned}$$

The domain of each layer ${\Omega }^{(k)}$ is then defined by

2.2 $$\begin{aligned} {\Omega }^{(k)} = \left\{ (x,y,z)\;|\;x\in \left[ 0;L\right] , (y,z) \in A^{(k)}\right\} \end{aligned}$$

### Material and mechanical properties

Laminated non-prismatic beams comprise multiple homogeneous layers stacked in a specific order. As such, the Young’s modulus of the entire beam “*E*(*x*, *z*)” can be expressed as a piecewise function

2.3 $$\begin{aligned} E(x,z)=\left\{ E^{(k)}\;|\;(x,z) \in {\Omega }^{(k)}\right\} \end{aligned}$$

Since the material properties vary through the cross-section, it is appropriate to include Young’s modulus in the generalization of stiffness properties. For example, the *longitudinal stiffness* is given by

2.4 $$\begin{aligned} \widetilde{A}(x)=\iint _{A(x)}E(x,z)\mathrm {d}A \end{aligned}$$

Noting that Eq. (2.4) agrees with the classical definition of longitudinal stiffness of homogeneous beams for the case where $n=1$. Analogous to $\widetilde{A}(x)$, the *first moment of stiffness* $\widetilde{S}(x)$ is defined as

2.5 $$\begin{aligned} \widetilde{S}(x)=\iint _{A(x)}E(x,z)z\mathrm {d}A \end{aligned}$$

Consequently, the neutral axis *c*(*x*) reads

2.6 $$\begin{aligned} c(x) = \frac{\widetilde{S}(x)}{\widetilde{A}(x)} \end{aligned}$$

Clearly, the stacking sequence plays an important role in defining the neutral axis position. Finally, the *bending stiffness* in relation to the neutral axis yields

2.7 $$\begin{aligned} \widetilde{I}(x)=\iint _{A(x)}E(x,z)\left( c(x)-z\right) ^2\mathrm {d}A \end{aligned}$$

The expressions introduced in this section are necessary to adapt essential concepts of homogeneous beam theories to heterogeneous media. However, Eqs. (2.4), (2.5) and (2.7) should not be interpreted as work conjugate of generalized stresses and strains but as stiffness-related variables that simplify the stress recovery procedure. It is noteworthy that curvature, axial, and shear deformations are coupled with all generalized stresses (Vu-Quoc and Léger 1992; Balduzzi et al. 2016, 2017a; Mercuri et al. 2020); hence constitutive relations between generalized strains and stresses are not readily extracted from application of compatibility relations to internal force definitions, as is common practice in prismatic beam theories. To overcome this impasse, previous works used the Complementary Virtual Work Principle (Vu-Quoc and Léger 1992), the Hellinger-Reissner Principle (Beltempo et al. 2015; Auricchio et al. 2015; Mercuri et al. 2020), and stress potential approaches (Balduzzi et al. 2016, 2017b). Since the proposed methodology is based on Newton’s Third Law, it only requires equilibrium relations (derived in the next section) and a definition for the longitudinal stress to recover the transverse stress components, which are expressed in terms of Eqs. (2.4)–(2.7).

### Equilibrium relations

**Fig. 2:**
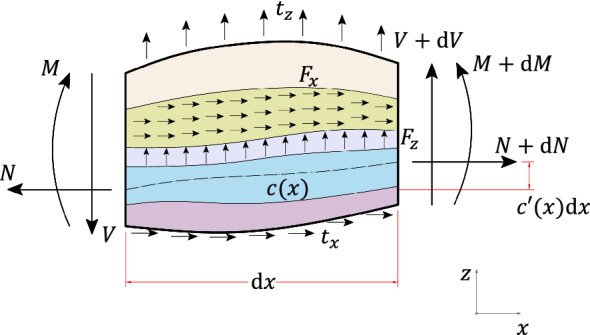
Laminated non-prismatic beam in equilibrium

The beam element is subject to layerwise body forces $F_i^{(k)}$ ($i=x,z$), uniformly distributed through a layer and variable spanwise, and to traction forces $t_i^{(k)}$ applied to $f^{(k)}$, as Fig. 2 depicts. The body force resultants in the longitudinal and transverse directions, namely $T_x$ and $T_z$, as well as the bending moment *m* originating from eccentric axial loading, are given by

2.8a $$\begin{aligned}&T_i(x) = \sum _{k=1}^{n+1}\left( \iint _{A(x)} F_i^{(k)}\mathrm {d}A + t_i^{(k)} \right) \;;\; i=(x,z) \end{aligned}$$

2.8b $$\begin{aligned}&m(x)=\sum _{k=1}^{n+1}\left( \iint _{A(x)} F_i^{(k)}\left( c-z\right) \mathrm {d}A + t_i^{(k)}\left( c-f^{(k)}\right) \right) \end{aligned}$$

The stress resultants are known variables but not necessarily evaluated from statically determinate models. The generalized longitudinal and shear stresses *N*(*x*) and *V*(*x*), respectively, and the generalized bending moment *M*(*x*) are given by the following expressions

2.9a $$\begin{aligned}&N(x)=\iint _{A(x)}\sigma _{xx}\mathrm {d}A \end{aligned}$$

2.9b $$\begin{aligned}&V(x)=\iint _{A(x)}\tau _{xz}\mathrm {d}A \end{aligned}$$

2.9c $$\begin{aligned}&M(x)=\iint _{A(x)}\sigma _{xx}\left( c-z\right) \mathrm {d}A \end{aligned}$$

where $\sigma _{xx}$ and $\tau _{xz}$ are the longitudinal and shear stress components, respectively.

From now on, the generalized bending moment *M*(*x*) is referred to as *bending moment* while the terminology *bending load* is adopted to refer to the bending moment *m*(*x*) caused by eccentric axial forces. Note that, even if the external load is reacted uniformly distributed through the cross-section, and no traction is prescribed, $m(x)\ne 0$ because the neutral axis position depends on the stiffness distribution, which is irrespective of the external load idealization.

Consider a segment of a laminated non-prismatic beam in equilibrium as illustrated in Fig. 2, where internal forces vary linearly along the infinitesimal length.

The Timoshenko equilibrium expressions are adapted to non-prismatic beam shapes by accounting for the neutral axis variation as follows

2.10a $$\begin{aligned}&\frac{\mathrm {d}N}{\mathrm {d}x}=-T_x \end{aligned}$$

2.10b $$\begin{aligned}&\frac{\mathrm {d}V}{\mathrm {d}x}=-T_z\end{aligned}$$

2.10c $$\begin{aligned}&\frac{\mathrm {d}M}{\mathrm {d}x} + V(x) - N(x)\frac{\mathrm {d}c}{\mathrm {d}x}=-m(x) \end{aligned}$$

The cross-sectional stiffness distribution does not affect the equilibrium of forces, but it influences the equilibrium of bending moments. For single layered beams, Eq. (2.10) simplifies to the equilibrium equations of homogeneous tapered beams (Vilar et al. 2021a; Balduzzi et al. 2016, 2017c, d).

### Interfacial boundary equilibrium

**Fig. 3:**
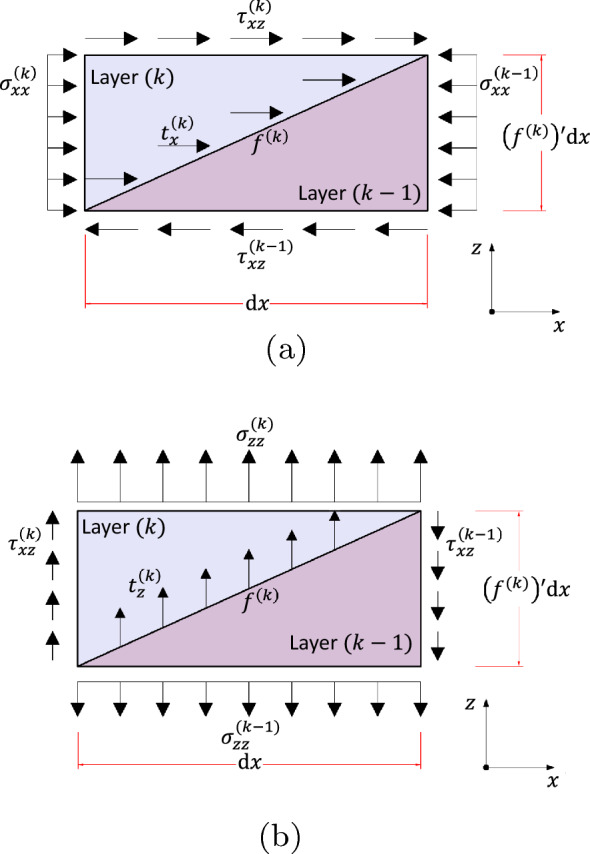
Interface equilibrium in: **a** *x*-direction, **b** *z*-direction

Consideration of equilibrium of forces requires specific relations between stress components at interfaces. One method to derive the interlayer boundary condition is to adapt the Cauchy stress equilibrium by selecting an appropriate representative unit cell, such as that illustrated in Fig. 3.

According to classic Cauchy stress equilibrium, an interior point of a 2D structure is represented by a rectangular shape of two independent infinitesimal dimensions. However, to best represent the interfacial boundary, the adopted unit cell dimensions are related by $f'^{(k)}$. Also, classic Cauchy stress equilibrium assumes the stress field varies linearly within an infinitesimal point, but this hypothesis does not hold for regions of discontinuous material properties. Instead, the stresses acting on the unit cell faces are independent variables.

Invoking the equilibrium of forces in the longitudinal and transverse directions shown in Fig. 3 yields

2.11a $$\begin{aligned}&{\Delta } \tau _{xz}^{(k)} = \frac{\mathrm {d}f}{\mathrm {d}x}^{(k)}{\Delta }\sigma _{xx}^{(k)}-t_x^{(k)}H_f^{(k)} \end{aligned}$$

2.11b $$\begin{aligned}&{\Delta } \sigma _{zz}^{(k)} = \frac{\mathrm {d}f}{\mathrm {d}x}^{(k)}{\Delta } \tau _{xz}^{(k)} -t_z^{(k)}H_f^{(k)} \end{aligned}$$

where $\sigma _{zz}$ is the transverse direct stress component and $H_f^{(k)}$ the hypotenuse of Fig. 3, and the ${\Delta }(.)^{(k)}$ operator indicates a discontinuity of its argument evaluated at interface surface $f^{(k)}$

2.12a $$\begin{aligned}&{\Delta }(.)^{(k)} = (.)^{(k)} - (.)^{(k-1)} \end{aligned}$$

2.12b $$\begin{aligned}&H_f^{(k)}=\sqrt{1+(f')^2} \end{aligned}$$

The second term of the right-hand side of Eq. (2.12a) vanishes for the first layer. It is noteworthy that the body force contribution to the interlayer force equilibrium is infinitesimal compared to the stress and traction resultants, and that one can substitute Eq. (2.11a) into Eq. (2.11b) to obtain the transverse interface boundary condition in terms of longitudinal stresses.

Equation (2.11) suggests that the transverse stresses are continuously distributed through the depth of the beam for traction-free prismatic beams, as reported in related studies (Bareisis 2006; Balduzzi et al. 2017a; Reddy 2003; Pagano 1978). Conversely, the transverse stress components are discontinuously distributed through the cross-section in non-prismatic beams due to interface surfaces being oblique to the global reference system (Balduzzi et al. 2017a).

It is relevant to highlight that the equilibrium derived in Eq. (2.11) is valid for an interior point of the cross-section over the interface boundary. Hence the traction-free boundary condition on the cross-section contour is not satisfied since the plane stress hypothesis is not valid in this region.

## Stress field recovery

This section introduces analytical expressions for the transverse stress field of laminated non-prismatic beams of general cross-sectional shapes. Closed-form solutions can be obtained from the analytical formulation by deriving the stiffness and geometric properties for specific cross-sectional shapes, which are given in the Appendix for the specific case of rectangular cross-sections.

The proposed method integrates the Cauchy stress equilibrium of an interior point in an arbitrary lamina, followed by imposition of interfacial boundary conditions derived in Sect. 2.3. As a result of the linear strain assumption coupled with a state of plane stress and material idealization, the longitudinal stress yields the well-known expression (Zenkert 1997)

3.1 $$\begin{aligned} \sigma _{xx}^{\left( k\right) }(x,z) = \left[ \frac{N(x)}{\widetilde{A}(x)} + M(x)\frac{\left( c(x)-z\right) }{\widetilde{I}(x)}\right] E^{\left( k\right) } \end{aligned}$$

Noting that the taper angle is limited to $15^{\circ }$ to reduce error of Eq. (3.1) (Boley 1963; Balduzzi et al. 2016; Vilar et al. 2021a).

### Shear stress

Cauchy stress equilibrium applied to an interior point of an arbitrary layer yields

3.2 $$\begin{aligned} \tau _{xz}^{(k)}(x,z) = -\iint _{\bar{A}}\left( \frac{\partial \sigma _{xx}^{(k)}}{\partial x} + F_x^{(k)} \right) \mathrm {d}A \end{aligned}$$

where $\bar{A}$ is an arbitrary slice of a cross-sectional area with an arbitrary *z* value for the upper boundary and undefined lower boundary. Information on the lower boundary coordinate is recovered by imposition of interface boundary conditions.

To simplify operations on Eq. (3.2), the vector ${\Gamma }$ containing terms of the equilibrium relations and their derivatives is introduced

3.3 $$\begin{aligned} {\Gamma } = \left\{ N \;(V+m)\; M\;N'\;V'\;m'\;N''\right\} ^T \end{aligned}$$

where $()'$ denotes differentiation.

Substituting for $\sigma _{xx}^{(k)}$ from Eq. (3.1) and the generalized external load and internal forces defined in Eqs. (2.8a) and (2.10) leads to the shear stress expressed as a linear combination as follows

3.4 $$\begin{aligned} \tau _{xz}^{(k)}(x,z) = \sum _{i=1}^{4}\left( \eta _i^{(k)}(x,z){\Gamma }_i(x)\right) + \kappa ^{(k)}_1(x) \end{aligned}$$

where $\kappa ^{(k)}_1$ is a measure of stress, constant in the *yz*-plane, as a result of the indefinite integration performed in Eq. (3.2). Expressions for $\eta ^{(k)}$ are given by

3.5 $$\begin{aligned} \begin{aligned}&\eta ^{(k)}_{N}=-E^{(k)}\left[ \frac{\partial }{\partial x}\left( \frac{\bar{A}}{\widetilde{A}}\right) + \frac{1}{\widetilde{I}}\frac{\mathrm {d}c}{\mathrm {d}x}\phi \right] \\&\eta ^{(k)}_{(V+m)}=E^{(k)}\phi \\&\eta ^{(k)}_{M}=-E^{(k)}\frac{\partial \phi }{\partial x}\\&\eta ^{(k)}_{N^{'}}= \left[ \frac{F_x^{(k)}}{T_x}-\frac{E}{\widetilde{A}}^{(k)}\right] \bar{A} \end{aligned} \end{aligned}$$

noting that the last term of the right hand side of (3.2) is expressed in terms of generalized longitudinal stress in the expressions for $\eta ^{(k)}_{N^{'}}$. The introduced variable $\phi$ is defined as

3.6 $$\begin{aligned} \phi =\iint _{\bar{A}}\left( \frac{c-z}{\widetilde{I}}\right) \mathrm {d}A \end{aligned}$$

Variables $\eta _i^{(k)}$ are defined as *longitudinal Cauchy stress coefficients*. These stiffness coefficients relate the internal forces to the shear stress distribution (e.g. $\eta _M^{(k)}$ relates bending moment to shear stress). As such, $\eta _i^{(k)}$ can be understood as intra-layer functions that recover the shear stress distribution from terms of the equilibrium relations and their derivatives.

The $\tau _{xz}$ dependency on all internal forces is referred to as the *non-triviality of stress field* in previous works (Vu-Quoc and Léger 1992; Hodges et al. 2011; Balduzzi et al. 2016, 2017a, d; Bruhns 2003; Vilar et al. 2021a). In addition to the internal forces and the bending load, $N'(x)$ contributes to the shear stress profile of laminated beams. In homogeneous media, $\eta _{N'}$ vanishes if the external load is generalized through the cross-section (Taglialegne 2018; Bertolini et al. 2019; Bertolini and Taglialegne 2020; Vilar et al. 2021a) because $F_x/T_x=F_x/(F_xA)=1/A$ is balanced by $-E/\tilde{A}=-E/(EA)=-1/A$ [see Eq. (3.5)]. Conversely, this balance does not occur in heterogeneous beams since the ratio $E^{(k)}/\tilde{A}$ could take any value from zero to one. Hence, in general, $\eta _{N'}^{(k)}$ does not vanish for laminated beams, and its effects should be considered for practical applications.

Apart from $\eta _V^{(k)}$, all longitudinal Cauchy stress coefficients carry information on cross-sectional variation and beam eccentricity. For single layered prismatic beams, Eq. (3.4) reduces to Jourawski’s formulation. This observation suggests that the classic solution for prismatic beams is part of the solution for tapered shapes (Vilar et al. 2021a, b).

The constant $\kappa ^{(k)}_1$ is evaluated by setting $z=f^{(k)}$ in Eq. (3.4), followed by imposition of the longitudinal interfacial condition stated in Eq. (2.11a)

3.7 $$\begin{aligned} \begin{aligned} \kappa ^{(k)}_1&=-\sum _{i=1}^{4}\left( \eta _i^{\left( k\right) }(x,f^{\left( k\right) }){\Gamma }_i(x) \right) + \frac{\mathrm {d}f}{\mathrm {d}x}^{(k)}{\Delta }\sigma _{xx}^{(k)} \\&+\tau _{xz}^{\left( k-1\right) }(x,f^{(k)}) \end{aligned} \end{aligned}$$

The expression for $\kappa ^{(k)}_1$ shown in Eq. (3.7) can be separated into two different groups:

1. the first term balances the Eq. (3.2) residual originating from integration of Cauchy stress equilibrium;
2. the remaining variables enforce longitudinal interfacial equilibrium.

Therefore, group (1) ensures the longitudinal interlayer boundary condition is recovered by group (2). For the first layer, if no traction is prescribed, $\kappa ^{(1)}_1$ coincides with the traction free boundary condition for homogeneous beams that is well-established in related research (Balduzzi et al. 2016, 2017a; Bleich 1932; Bruhns 2003; Hodges et al. 2011; Vilar et al. 2021a, b; Vu-Quoc and Léger 1992).

The constant $\kappa ^{(k)}_1$ requires information on the lower adjacent layer to define the shear stress. For instance, $\tau _{xz}^{(2)}$ profile can be determined only if $\kappa ^{(1)}_1$ and $\tau _{xz}^{(1)}$ have already been evaluated. As such, after performing successive substitutions from the *k*th to the bottom layer, the shear stress profile is expressed as follows

3.8 $$\begin{aligned} \tau _{xz}^{(k)}=\sum _{l=1}^{k}\left( \sum _{i=1}^{4}\left( \left[ \eta _i^{(k)} -{\Delta }\eta _i^{(l)}\right] {\Gamma }_i\right) +{\Delta }\tau _{xz}^{(l)}\right) \end{aligned}$$

Note that the successive substitution process is considered by the summation operator from dummy index *l* varying from one to the *k*th layer.

The shear stress given by Eq. (3.8) is novel and more accurate than related analytical expressions reported in up-to-date literature (Balduzzi et al. 2017a). The key novelties regarding $\tau _{xz}$ derived include consideration of: the longitudinal traction and body forces, both defined for each layer; the beam eccentricity; the first derivative of the generalized longitudinal stress.

### Transverse direct stress

Analogous to the methodology derived in Sect. 3.1, Cauchy stress equilibrium is invoked in the transverse direction

3.9 $$\begin{aligned} \sigma _{zz}^{(k)} = -\iint _{\bar{A}}\left( \frac{\partial \tau _{xz}^{(k)}}{\partial x} + F_z^{(k)} \right) \mathrm {d}A \end{aligned}$$

Substituting the expression for $\tau _{xz}^{(k)}$ given by Eq. (3.4) and the generalized external load and internal forces defined in Eqs. (2.8a) and (2.10), yields

3.10 $$\begin{aligned} \sigma _{zz}^{(k)}=\sum _{j=1}^7\left( \zeta _j^{(k)}{\Gamma }_j\right) - \frac{\mathrm {d}}{\mathrm {d}x}\left( \kappa ^{(k)}_1\right) \bar{A} + \kappa _2^{(k)} \end{aligned}$$

where $\kappa _2^{(k)}$ is a constant value of stress in the cross-section as a result of indefinite integration of Eq. (3.9).

Expressions for $\zeta _j^{(k)}$ are given by

3.11 $$\begin{aligned} \begin{aligned}&\zeta _i^{(k)}(x,z) = -\iint _{\bar{A}}\lambda _i^{(k)}(x,z)\mathrm {d}A \\&\lambda _N^{(k)}=\frac{\partial \eta _N^{(k)}}{\partial x} + \frac{\mathrm {d}c}{\mathrm {d}x}\eta _M^{(k)} \\&\lambda _{(V+m)}^{(k)}=\frac{\partial \eta _{(V+m)}^{(k)}}{\partial x} - \eta _M^{(k)} \\&\lambda _{M}^{(k)}=\frac{\partial \eta _M^{(k)}}{\partial x} \\&\lambda _{N^{'}}^{(k)}=\frac{\partial \eta _{N^{'}}^{(k)}}{\partial x} + \eta _N^{(k)} \\&\lambda _{V^{'}}^{(k)}= \eta _{(V+m)}^{(k)} -\frac{F_z^{(k)}}{T_z}\bar{A} \\&\lambda _{m^{'}}^{(k)}=\eta _{(V+m)}^{(k)} \\&\lambda _{N^{''}}^{(k)}=\eta _{N^{'}}^{(k)} \end{aligned} \end{aligned}$$

Note that the dummy index in the summation operator of Eq. (3.10) varies from $j=(1,2,\ldots ,7)$, representing a range of values that differs from that in Eq. (3.4) because the transverse direct stress requires all terms of ${\Gamma }$ (see Eq. (3.3)). As such, the transverse direct stress depends on all internal forces, the bending load, and their derivatives (up to the second derivative for the case of the generalized longitudinal stress).

The constant $\kappa _2^{(k)}$ is deduced by invoking the transverse interlaminar boundary condition expressed in Eq. (2.11b)

3.12 $$\begin{aligned} \begin{aligned} \kappa ^{(k)}_2&=-\sum _{j=1}^{7}\left( \zeta _j^{(k)}{\Gamma }_j\right) + \frac{\mathrm {d}}{\mathrm {d}x}\left( \kappa _1^{(k)}\right) \bar{A}\\&\quad +\frac{\mathrm {d}f}{\mathrm {d}x}^{(k)}{\Delta }\tau _{xz}^{(k)} +\sigma _{zz}^{\left( k-1\right) } \end{aligned} \end{aligned}$$

Note that $z=f^{(k)}$ has been omitted for $\bar{A}$ and $\zeta _j$. Analogous to $\kappa _1^{(k)}$, variable $\kappa _2^{(k)}$ can be characterized by different contributions:

1. the first term balances the Eq. (3.9) residual that arises due to indefinite integration;
2. the last two terms ensure transverse interlayer equilibrium;
3. the term involving $\kappa _1^{(k)}$ arises from mathematical operations on Eq. (3.9) regarding longitudinal interlayer equilibrium.

Following an analogous procedure of successive substitutions explained in Sect. 3.1, an analytical expression for the transverse direct stress is obtained

3.13 $$\begin{aligned} \begin{aligned} \sigma _{zz}^{(k)}&=\sum _{l=1}^{k}\Bigg (\sum _{j=1}^{7}\left( \left[ \zeta _j^{(k)} - {\Delta }\zeta _j^{(l)} +{\Delta }\lambda _j^{(l)}\hat{A}^{(l)}\right] {\Gamma }_j\right) \\&\quad -\frac{\mathrm {d}}{\mathrm {d}x}\left( {\Delta }\tau _{xz}^{(l)}\right) \hat{A}^{(l)} +{\Delta }\sigma _{zz}^{(l)}\Bigg ) \end{aligned} \end{aligned}$$

where the dependence of $\zeta _j^{(k)}$ on (*x*, *z*) and the ${\Delta }(.)$ operators on $(x,f^{(k)})$ have been omitted. The introduced variable $\hat{A}^{(l)}$ is defined by

3.14 $$\begin{aligned} \hat{A}^{(l)} = \bar{A}(x,z)-\bar{A}(x,f^{(l)}) \end{aligned}$$

The transverse direct stress expressed by Eq. (3.13) is new and applies to laminated tapered beams under layerwise body loads and traction forces, considering the simplifications discussed in Sect. 2.

## Numerical results

This section solves numerical examples to discuss and validate the proposed methodology. To benchmark the developed theory, results are compared to finite element analysis solutions, which are referred to as “FEA” in the next section. The current formulation is labeled as “mod”, whose longitudinal and shear stress distributions are compared with Balduzzi et al.’s (2017a) solution, denoted as “Bal”. The differences between Balduzzi et al.’s (2017a) shear stress to that derived in Eq. (3.8) concern the neglect of beam eccentricity and bending load, as well as the assumption that the external load is generalized through the cross-section. To the best of the authors’ knowledge, no other analytical solution for $\sigma _{zz}$ has been derived for laminated tapered beams.

The following beam models are solved considering a rectangular cross-section:

1. a symmetrically tapered sandwich cantilever subject to a transverse concentrated force at the tip;
2. a simply supported double tapered beam under a transverse body force applied to a thin layer;
3. an asymmetric cantilever beam with the upper surface flat under:
  (3.1) a longitudinal uniform body force;
  (3.2) constant traction and pressure loads applied to the upper surface.

The terminology “case (.)” is used to refer to the beam models in this section.

A mesh refinement study was performed to ensure the finite element analyses converged, noting that the number of elements between mesh refinements is multiplied by a factor of 1.5–2.5. Relative errors defined as $\epsilon _i=(\sigma _{zz}^{(i)} - \sigma _{zz}^{(i-1)})/\sigma _{zz}^{(i)}$ were calculated between mesh refinements at a specific point of the beam domain (index *i* refers to the *i*th mesh refinement). Finite element analysis results were considered to be converged when $\epsilon _i\le 0.02\%$. Finally, the relative error of the proposed methodology in relation to the converged finite element mesh is given by $\epsilon _{mod}=(\sigma _{zz}^{(mod)} - \sigma _{zz}^{(i)})/\sigma _{zz}^{(mod)}$ (*mod* concerns the developed model). Note that $\epsilon _{mod}$ should not be interpreted as a measure of accuracy of the proposed model because it is evaluated at a specific point. The 8-node quadrilateral plane stress element (CPS8R in Abaqus terminology) was used for cases (1) and (3) while the 6-node triangular plane stress element (CPS6R) was used for case (2). More information on the mesh structure is given in the respective model discussion. In each case, the processing time has been calculated to compare performance, noting that the proposed formulation was coded in Matlab2019a. The computer used for both analyses was a model i7-6800 CPU @ 3.40GHz with 32 GB RAM installed.

### Linearly symmetric

**Fig. 4:**
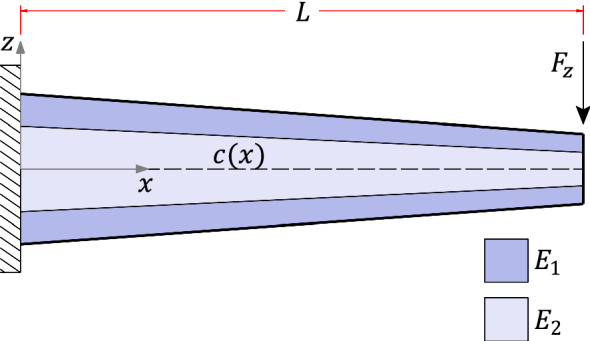
Case (1) boundary conditions

Consider the laminated cantilever symmetric beam illustrated in Fig. 4, of length $L={10}{\hbox { m}}$ and width $w={1}{\hbox { m}}$. Due to its symmetry in the *x*-direction, this model has zero beam eccentricity. As such, the aim of case (1) is to test the proposed formulation for the specific case of $c'(x)=0$.

This beam has outer layers of Young’s modulus $E^{(1)}=E^{(3)}={800}{\hbox { GPa}}$ separated by a softer core $E^{(2)}={50}{\hbox { GPa}}$. The following interface surfaces are set for this model (in mm)

4.1 $$\begin{aligned} {\left\{ \begin{array}{ll} \begin{aligned} &f^{(1)}= - f^{(4)} = \frac{3}{64}x -625\\ &f^{(2)}= - f^{(3)} = \frac{9}{320}x -375 \end{aligned} \end{array}\right. } \end{aligned}$$

The thickness of the core is double that of the outer layers, and the beam depth at the fixed end is $h(0)={1.25}\hbox { m}$, while at $h(L)={0.625}\hbox { m}$, resulting in a constant $1.79^{\circ }$ taper angle.

The beam is subject to a concentrated force at the tip such that $T_z(L)={-1}{\hbox { kN}}$, resulting in the internal forces at cross-sections $x=0.25L$ and $x=0.90L$ given in Table 1.

**Table 1:** Case (1) internal forces

*x*/*L* (–)	*V* kN	*M* kNm
0.25	$-1$	$-7.5$
0.90	$-1$	$-1$

**Fig. 5:**
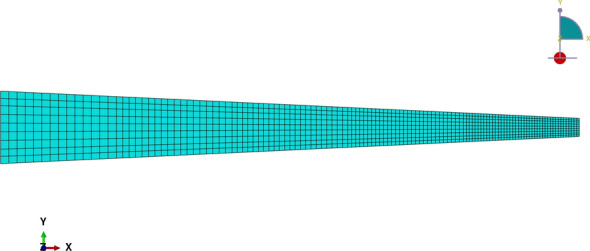
Case (1) typical mesh

A typical mesh layout is shown in Fig. 5; the number of elements through the thickness is constant, resulting in an average aspect ratio of 1.07 for the converged mesh. Values for $\sigma _{zz}$ are evaluated at the top surface of the cross-section at $x=0.25L$ for the mesh convergence analysis given in Table 2. The adopted Poisson’s ratio was $\nu =0.25$ for all layers. The processing time of the finite element analysis was 5.6 s using Abaqus software against 0.053 s from the proposed method.

**Table 2:** Case (1) mesh convergence analysis

Number elements	Av. aspect ratio	$\sigma _{zz}$ (MPa)	$\epsilon _{i}$ ($\%$)	$\epsilon _{mod}$ ($\%$)
14,539	1.08	0.12010	–	$-0.08$
22,191	1.07	0.12024	0.116	0.03
35,966	1.07	0.12026	0.017	0.05

**Fig. 6:**
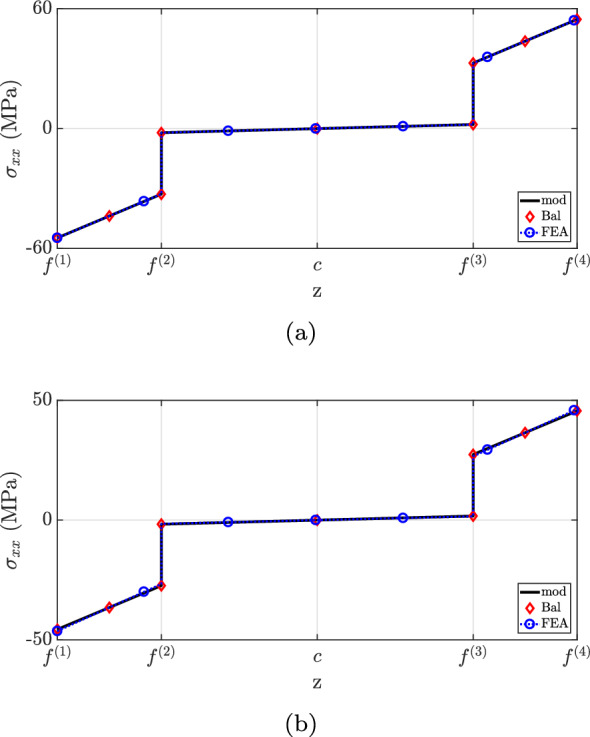
$$\sigma _{xx}$$ for case (1): **a** $$x=0.25L$$, **b** $$x=0.90L$$

The longitudinal stress field is shown in Fig. 6. Notably, all methodologies result in the same distribution. The magnitude of $\sigma _{xx}$ at the outer layers is significantly greater than at the core due to their larger Young’s modulus. Moreover, the discontinuity at $f^{(2)}$ and $f^{(3)}$ represent the sudden change in elastic properties on the outer-layer/core interfaces.

**Fig. 7:**
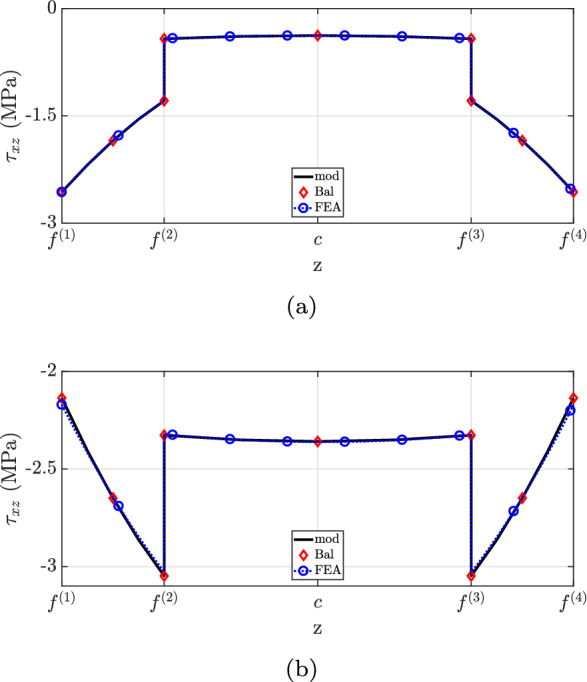
$$\tau _{xz}$$ for case (1): **a** $$x=0.25L$$, **b** $$x=0.90L$$

Figure 7 depicts the results for $\tau _{xz}$. Remarkably, all solutions provide the same parabolic distribution for both cross-sections. A larger discrepancy is observed between the finite element analysis and the current formulation for $x=0.90L$ due to localized effects closer to the applied load. The maximum absolute shear stress magnitude is located at the beam surfaces for $x=0.25L$ and the outer-layer/core interfaces for $x=0.90L$. This observation contradicts Jourawski’s solution for prismatic beams and highlights the non-triviality of the shear stress distributions. Additionally, the shear stress discontinuity at interfaces confirms the interface boundary equilibrium discussed in Sect. 2.3.

**Fig. 8:**
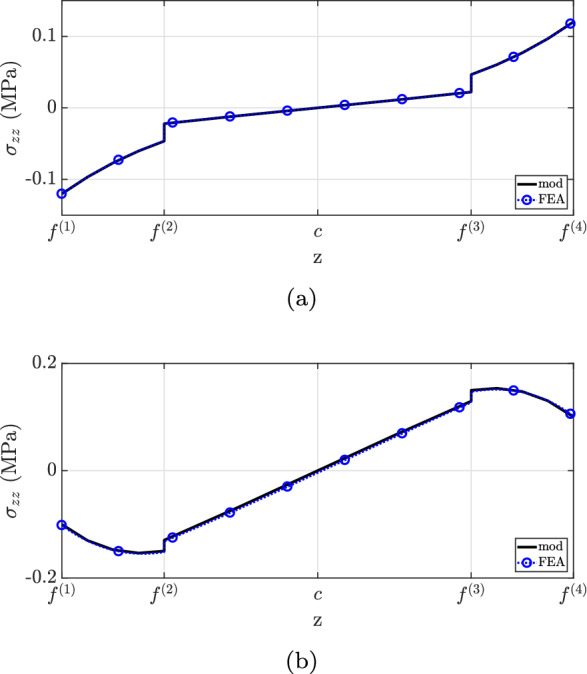
$$\sigma _{zz}$$ for case (1): **a** $$x=0.25L$$, **b** $$x=0.90L$$

The transverse direct stress is illustrated in Fig. 8, which shows that the solutions from the current formulation and that of finite element analysis match each other for both cross-sections. A small discrepancy between the two formulations is observed at $x=0.90L$ due to localized Saint Venant’s effects. Note that, the maximum $\sigma _{zz}$ occurs at the same location as maximum $\tau _{xz}$, i.e. at the beam surfaces for $x=0.25L$ and at the outer-layer/core interfaces for $x=0.90L$. It is relevant to highlight that the discontinuities at the interfacial surfaces for the case of $\sigma _{zz}$ are smaller than that for $\tau _{xz}$ because ${\Delta }\sigma _{zz}=f'{\Delta }\tau _{xz}$ and the small taper angle assumption drives $f'(.)\le 0.268$. Consequently, more significant stress discontinuities are expected in $\sigma _{zz}$ rather than in $\tau _{xz}$ for the case of $f'{(.)}>1$, which corresponds to a local taper angle larger than $45^{\circ }$, noting that this magnitude is an unlikely circumstance for most beam applications.

### Double-taper

**Fig. 9:**
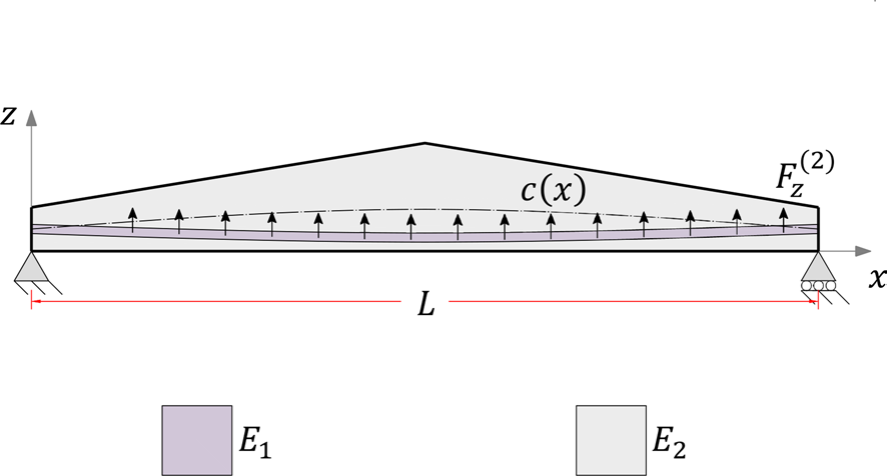
Case (2) boundary conditions

The section solves the beam model illustrated in Fig. 9. Case (2) consists of a simply supported double-tapered beam comprising two different materials: a ${30}\hbox { mm}$ stiffer layer of Young’s modulus $E^{(2)}={210}{\hbox { GPa}}$ embedded in softer layers with $E^{(1)}=E^{(3)}={25}{\hbox { GPa}}$. Moreover, the beam has $L={10}\hbox { m}$ and $w={1}\hbox { m}$.

The depth of the beam varies linearly over its span such that $h(0)=h(L)={0.5}\hbox { m}$ and $h(0.5L)={1}\hbox { m}$. Consequently, the taper angle is $9.09^{\circ }$ for the left half of the beam and $-9.09^{\circ }$ for the right half. The interfacial surfaces of case (2) are given by the following expressions (in mm)

4.2 $$\begin{aligned} {\left\{ \begin{array}{ll} \begin{aligned} &f^{(1)}=0\\ &f^{(2)} = \frac{16}{5}x^2 - 32x + 85 \\ &f^{(3)} = f^{(2)} + 30 \\ &f^{(4)} = 40\left( 45-4x\right) \;|\;x\le L/2 \\ &f^{(4)} =40\left( 4x+5\right) \;|\;x>L/2 \end{aligned} \end{array}\right. } \end{aligned}$$

This numerical example highlights a practical problem in engineering employed in building construction. Figure 9 could represent an application of prestressed concrete, which consists of highly-tensioned robust steel bars with a curved shape (Gilbert and Mickleborough 1990). According to concrete design codes (ACI Committee and International Organization for Standardization 2008; EN 2004), steel bar stiffening effects can be disregarded for design purposes. To give an insight into the error associated with this simplification, results for the specific case where the Young’s modulus $E={25}{\hbox { GPa}}$ is constant through the thickness are provided (referred to as “mod $(E^{(2)}=E^{(1)})$”).

The beam is subject to a distributed body force $F_z^{(2)}={1.111}{(10^4\,\hbox { kN}/\hbox {m}^3)}$ applied to the stiffest layer such that the transverse resultant over a cross-section is constant spanwise $T_z={333.33}{\hbox { kN}/\hbox {m}}$. Table 3 reports the internal forces and their derivatives for $x=0.25L$ and $x=0.60L$. It is relevant to mention that the current formulation is not able to predict the stress field at the midspan due to stress-channeling effects (Everstine and Pipkin 1971). Instead a higher-order theory that allows non-linear distribution of axial stress through-the thickness would be required, as for example shown by Groh and Weaver (2015) for the case of prismatic beams, and will be considered in future work.

**Table 3:** Case (2) internal forces

*x*/*L* (–)	*V* kN	*M* kNm	$V'$ kN/m
0.25	833.33	$-3125$	$-333.33$
0.60	$-333.33$	$-4000$	$-333.33$

**Fig. 10:**
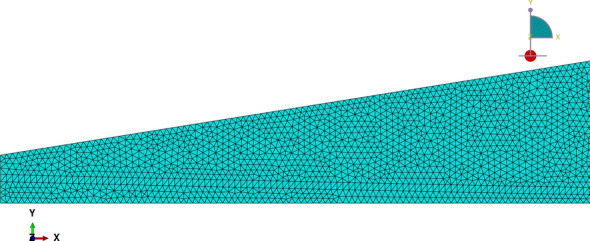
Case (2) typical mesh

In the finite element analysis, each layer of the beam has a different mesh arrangement, which follows a structured pattern only for the second layer, as illustrated in Fig. 10. Consequently, the mesh density “$\rho ^{k}$” varies through the thickness but is approximately uniform spanwise (where “*k*” indicates the layer index). More specifically, the ratio between mesh densities corresponds to $\rho ^{1}/\rho ^{2}=1.5$ and $\rho ^{1}/\rho ^{3}=3$. The mesh refinement study is given in Table 4, with values of $\sigma _{zz}$ calculated at the upper surface of $x=0.25L$. The adopted Poisson’s ratio was $\nu ^{(1)}=\nu ^{(3)}=0.3$ and $\nu ^{(2)}=0.2$. It is noteworthy that the choice for triangular elements arose from the curved geometry of the second layer. The processing time of the finite element analysis was 25.5s against 0.041s from the proposed method.

**Table 4:** Case (2) mesh convergence analysis

Number elements	Av. aspect ratio	$\sigma _{zz}$ (MPa)	$\epsilon _{i}$ ($\%$)	$\epsilon _{mod}$ ($\%$)
78,728	1.19	1.02201	–	$-0.031$
123,922	1.18	1.02211	0.010	$-0.21$
269,549	1.17	1.02217	0.006	0.015

**Fig. 11:**
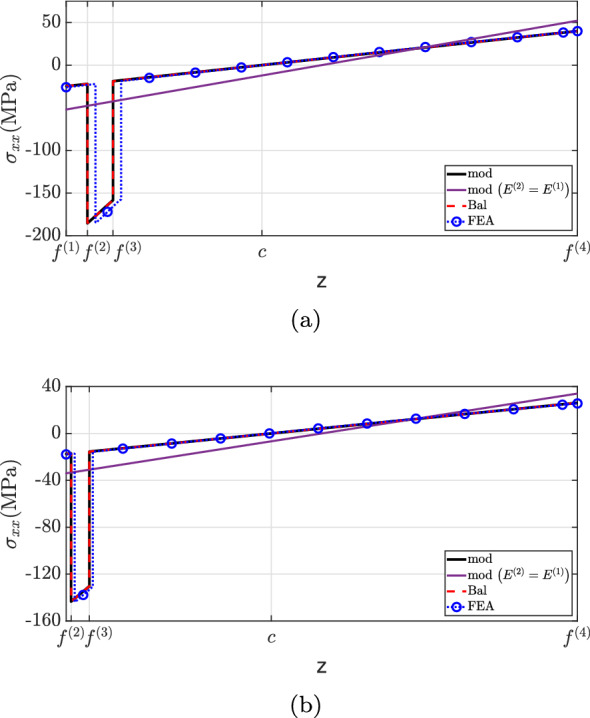
$$\sigma _{xx}$$ for case (2): **a** $$x=0.25L$$, **b** $$x=0.60L$$

The $\sigma _{xx}$ distribution is shown in Fig. 11. For both cross-sections, the current methodology matches Balduzzi et al.’s formulation (Balduzzi et al. 2017a) and the finite element analysis. However, a greater discrepancy is observed between the proposed theory and “FEA” results for $x=0.25L$ between $f^{(2)}$ and $f^{(3)}$. More specifically, the locations of maximum stress do not agree but their magnitudes are identical. The simplification suggested by design codes (ACI Committee and International Organization for Standardization 2008; EN 2004) fails to capture the correct neutral axis position and stress discontinuities at material interfaces, resulting in significant underestimation of $\sigma _{xx}$ for the stiffer layer.

**Fig. 12:**
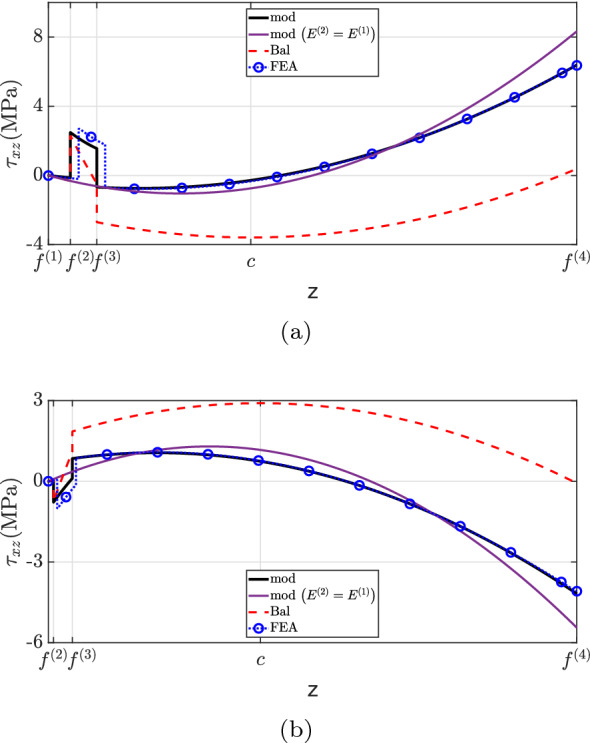
$$\tau _{xz}$$ for case (2): **a** $$x=0.25L$$, **b** $$x=0.60L$$

Figure 12 depicts the shear stress distribution. Notably, the proposed theory matches well with the finite element analysis, but discrepancies were observed at the second layer for both cross-sections. This inconsistency is explained by the neglect of Poisson’s ratio effects in the current method. State-of-the-art formulations have not captured the shear stress distribution accurately due to the neglect of beam eccentricity (“Bal”) and stiffer layer properties (“mod $(E^{(2)}=E^{(1)})$”). Regarding the traction-free boundary requirement, all models successfully predict the vanishing of shear stress on the flat surface, but only the developed formulation matched the finite element analysis at the top surface.

**Fig. 13:**
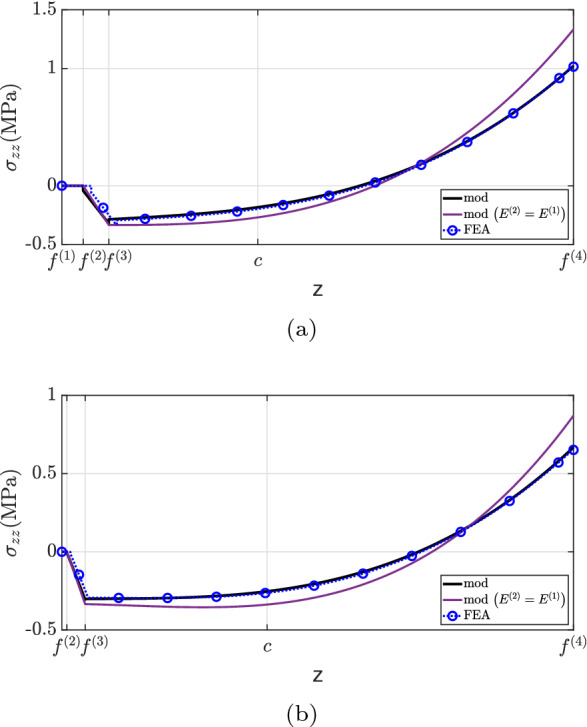
$$\sigma _{zz}$$ for case (2): **a** $$x=0.25L$$, **b** $$x=0.60L$$

Finally, the transverse direct stress field is shown in Fig. 13. The developed formulation matches well with the finite element analysis solution. Overall, a greater discrepancy is observed for $x=0.60L$. Furthermore, the simplification considered by assuming $E^{(1)}=E^{(2)}$ does not capture the $\sigma _{zz}$ distribution well and overestimates the transverse direct stress at the top surface. Similar to the previous example, the maximum absolute $\sigma _{zz}$ magnitude occurs at the same position as the maximum shear stress, i.e., at the top surface.

### Linearly asymmetric

Case (3) refers to the 4-layer cantilever beam of length $L={10}\hbox { m}$ and width $w={1}\hbox { m}$ illustrated in Figs. 15 and 19. The layers are made of different materials such that $E^{(1)}=2E^{(2)}=4E^{(3)}=8E^{(4)}={600}{\hbox { GPa}}$. Throughout the beam, all layers have the same thickness, with $h(0)={1}\hbox { m}$ at the fixed end and $h(L)={0.5}\hbox { m}$ at the free one, resulting in $2.862^{\circ }$ taper angle and the following interface functions (in mm)

4.3 $$\begin{aligned} {\left\{ \begin{array}{ll} \begin{aligned} &f^{(1)}=\frac{1}{20}x \\ &f^{(2)} =\frac{3}{80}x + 250 \\ &f^{(3)} =\frac{1}{40}x + 500 \\ &f^{(4)} =\frac{1}{80}x + 750 \\ &f^{(5)} =1000 \end{aligned} \end{array}\right. } \end{aligned}$$

For this example, two different loading conditions are considered:

- case (3.1): a constant longitudinal body force applied to the entire beam domain;
- case (3.2): constant traction and pressure loads acting on the horizontal surface.

**Fig. 14:**
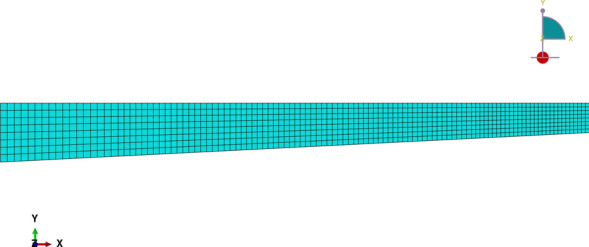
Case (3) typical mesh

The cross-sections $x=0.25L$ and $x=0.75L$ have been selected to compare results due to their remote distance from localized effects occurring near boundaries in the finite element analysis. A typical finite element mesh for case (3) is illustrated in Fig. 14; note that the number of elements in the thickness direction is constant spanwise. The convergence study is reported in Table 5, with $\sigma _{zz}$ values evaluated at the lower surface of cross-section $x=0.25L$ for the refinement analysis (Table 5$\sigma _{zz}$ values refer to case (3.2)). The processing time of the finite element analyses were 19 s and 19.6 s against 0.067 s and 0.063 s from the proposed method for cases (3.1) and (3.2), respectively.

**Table 5:** Case (3) mesh convergence analysis

Number elements	Av. aspect ratio	$\sigma _{zz}$ (kPa)	$\epsilon _{i}$ ($\%$)	$\epsilon _{mod}$ ($\%$)
32,016	1.19	904.11	–	0.13
76,076	1.19	903.873	$-0.026$	$-0.10$
133,400	1.19	903.856	$-0.002$	$-0.10$

#### Case (3.1)

**Fig. 15:**
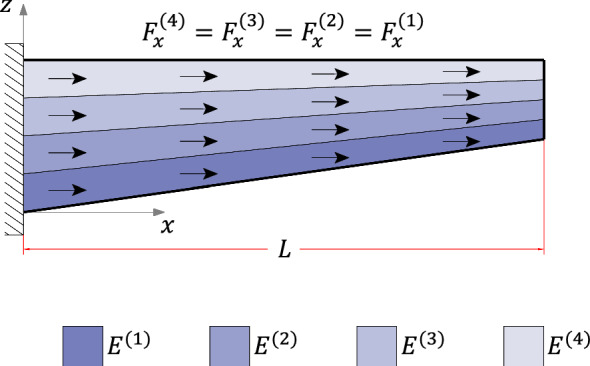
Case (3.1) boundary conditions

Consider the linearly asymmetric tapered beam with a longitudinal body force $F_x={1}{\hbox { kN}/\hbox {m}^3}$ applied to all layers, as shown in Fig. 15. The body force resultant is a linear function of the *x*-coordinate given by $T_x=1-0.05x$ (kN/m). The motivation to solve this load case is to validate the proposed formulation when $N''\ne 0$, a variable necessary to define the transverse direct stress field. Furthermore, a longitudinal body force applied to a slender structure is a typical load case of beams under centrifugal forces, which occurs in wind turbine blades and helicopter rotor blades.

Approximate results for the internal forces of case (3.1) and their derivatives are reported in Table 6.

**Table 6:** Case (3.1) internal forces

*x*/*L* (–)	*N* kN	*m* Nm/m	*M* kNm	$N'$ kN/m	$m'$ N/mm	$N''$ Pa
0.25	5.17	$-146.7$	$-1.30$	$-0.88$	0.02	50
0.75	1.41	$-74.5$	$-0.211$	$-0.63$	0.01	50

**Fig. 16:**
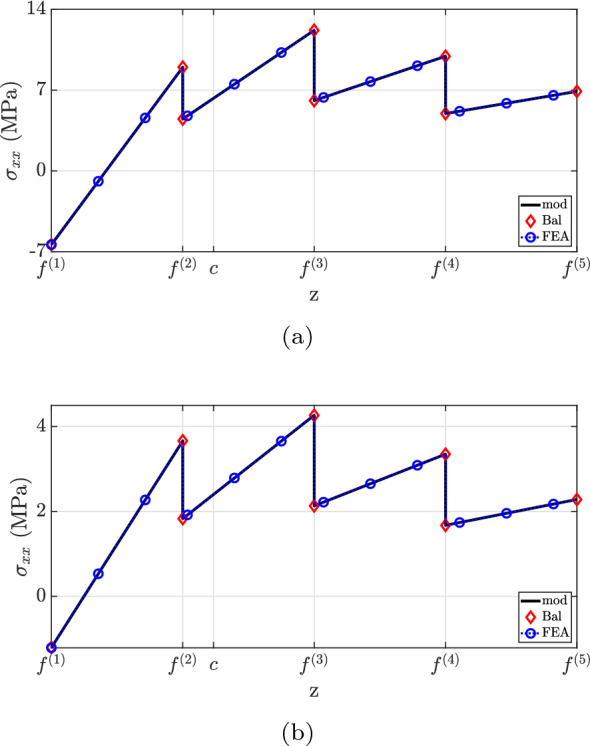
$$\sigma _{xx}$$ for case (3.1): **a** $$x=0.25L$$, **b** $$x=0.75L$$

The longitudinal stress results suggest that all solutions match the same piecewise linear distribution depicted in Fig. 16. As expected, the gradient of $\sigma _{xx}$ decreases through the thickness as a result of Young’s modulus reduction from the bottom to the top, noting that a similar trend is anticipated for the other stress components. Due to this particular material stiffness distribution, the centroid is located closer to the lower surface.

**Fig. 17:**
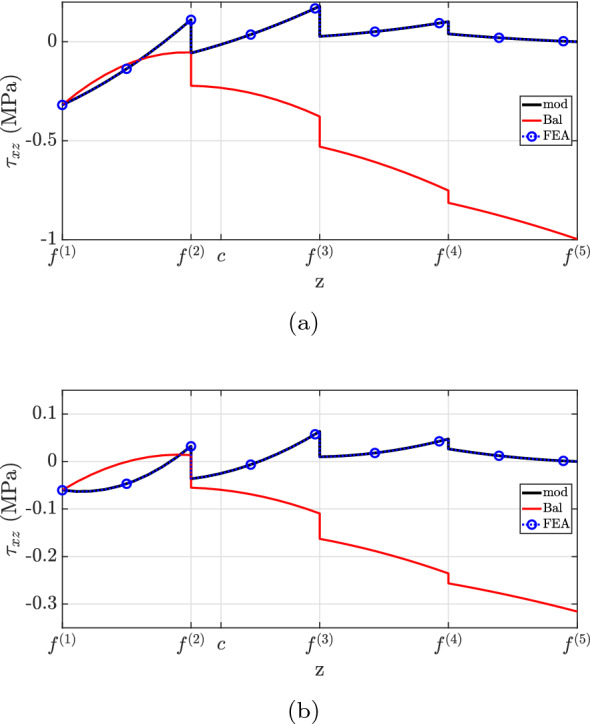
$$\tau _{xz}$$ for case (3.1): **a** $$x=0.25L$$, **b** $$x=0.75L$$

Figure 17 shows that the proposed model matches the shear stress distribution of the finite element analysis. Notably, the curvature of $\tau _{xz}$ is more pronounced for the cross-section at $x=0.75L$ rather than at $x=0.25L$, which occurs because the contributions of the parabolic terms, i.e., *N*(*x*), *M*(*x*) and *m*(*x*), when added together, are more significant at $x=0.75L$ compared to $x=0.25L$. In turn, the contribution of $N'(x)$, a linear function through the thickness, is accentuated at $x=0.25L$. The solution given by Balduzzi et al. (2017a) does not capture the appropriate shear distribution because of the neglect of beam eccentricity, bending load, and $N'(x)$. It is noteworthy that the maximum absolute shear stress occurs at the lower surface for $x=0.25L$ and at the interface between the second and the third laminae for $x=0.75L$. Moreover, the current formulation successfully captures the traction-free boundary conditions at both surfaces.

**Fig. 18:**
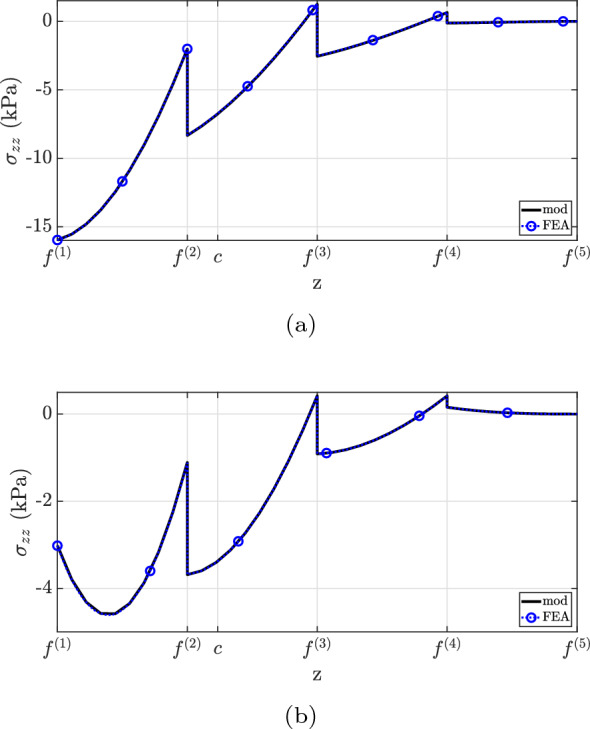
$$\sigma _{zz}$$ for case (3.1): **a** $$x=0.25L$$, **b** $$x=0.75L$$

Figure 18 depicts the excellent agreement between the proposed methodology and the finite element analysis for the transverse direct stress. The largest discrepancy observed was $0.1\%$, located at the lower surface, as reported in Table 5. Notably, the maximum absolute $\sigma _{zz}$ occurs at the lower surface for $x=0.25L$. In contrast, the maximum $\sigma _{zz}$ magnitude at $x=0.75L$ resides within the first layer, which is a different position from that of maximum shear stress. Additionally, the current formulation successfully captures the traction-free boundary conditions at the lower surface and the vanishing shear stress at the horizontal surface.

#### Case (3.2)

**Fig. 19:**
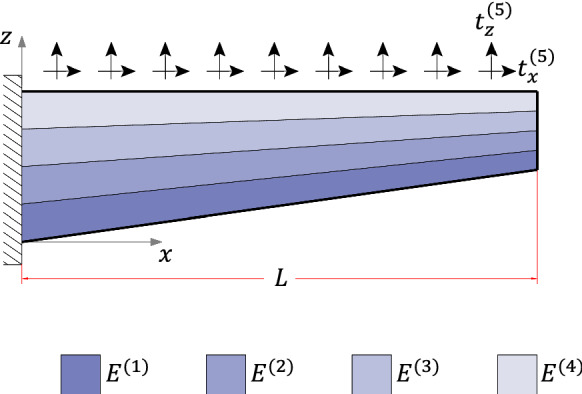
Case (3.2) boundary conditions

The loading case (3.2) illustrated in Fig. 19 is the linearly tapered beam of the former example subject to a traction force $t_x^{(5)}={1}{\hbox { kN}/\hbox {m}}$ and an outwards pressure load $t_z^{(5)}={1}{\hbox { kN}/\hbox {m}}$, both applied to the horizontal surface, resulting in the internal forces given in Table 7.

**Table 7:** Case (3.2) internal forces

*x*/*L* (–)	*N* kN	$V+m$ kN	*M* kNm	$N'=V'$ kN/m	$m'$ kN/m
0.25	7.5	6.895	23.586	$-1$	0.035
0.75	2.5	2.068	2.044	$-1$	0.035

**Fig. 20:**
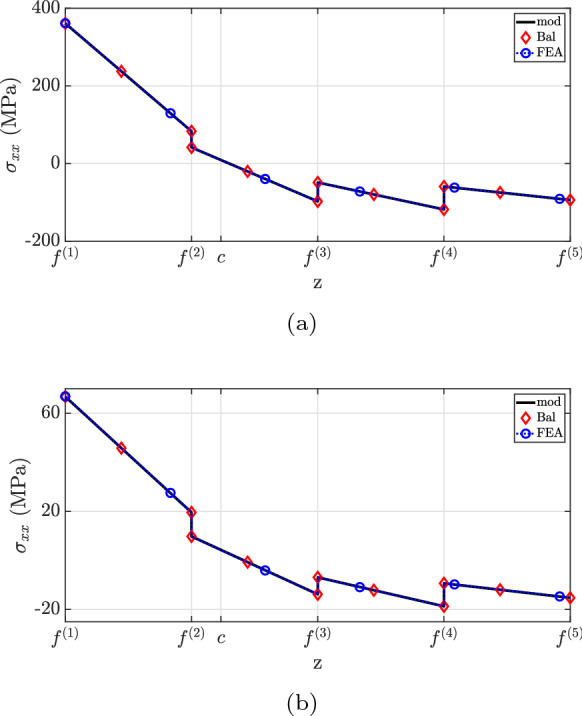
$$\sigma _{xx}$$ for case (3.2): **a** $$x=0.25L$$, **b** $$x=0.75L$$

The solutions for the longitudinal stress field shown in Fig. 20 result in the same piecewise linear distribution for all methodologies. Similar to the previous load case, the gradient of $\sigma _{xx}$ is proportional to the stiffness distribution, which is more significant for the lower layers. Note that the neutral axis position is closer to the centroid for cross-section $x=0.25L$ rather than at $x=0.75L$ because the bending moment is more pronounced in relation to the generalized longitudinal stress for $x=0.25L$.

**Fig. 21:**
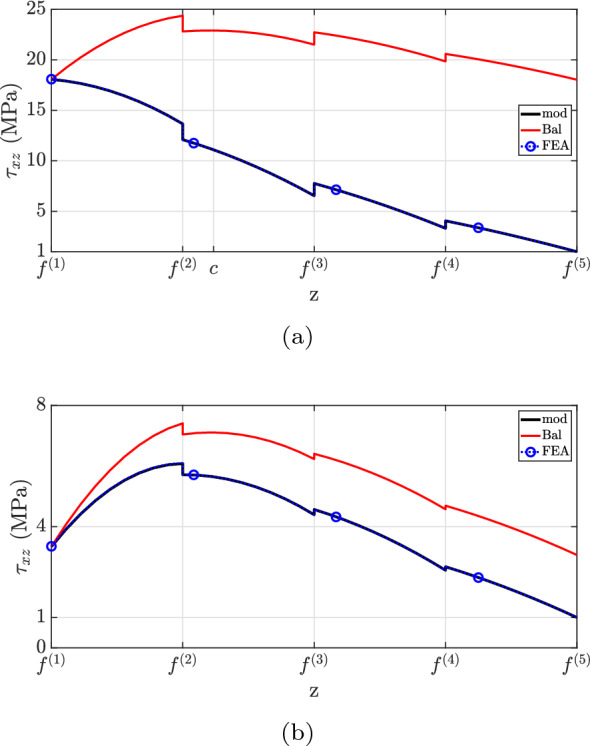
$$\tau _{xz}$$ for case (3.2): **a** $$x=0.25L$$, **b** $$x=0.75L$$

The shear stress prediction of the current methodology matches that of the finite element analysis, as depicted in Fig. 21. Notably, the analytical solution “Bal” has not captured the correct $\tau _{xz}$ distribution due to three different reasons: the neglect of beam eccentricity and bending load, and the assumption of external load being generalized to the entire cross-section. The appropriate traction requirement has been successfully captured by the developed methods ($\tau _{xz}={1}{\hbox { MPa}}$ at $z=f^{(5)}$ for both cross-sections). With regards to the $\tau _{xz}$ profile, the analyzed cross-sections exhibit different trends. The curvature of $\tau _{xz}$ is more pronounced for $x=0.75L$ rather than $x=0.25L$. Furthermore, the maximum absolute $\tau _{xz}$ magnitude is located at the lower surface for $x=0.25L$ whereas at the interface between the first and second layers for $x=0.75L$.

**Fig. 22:**
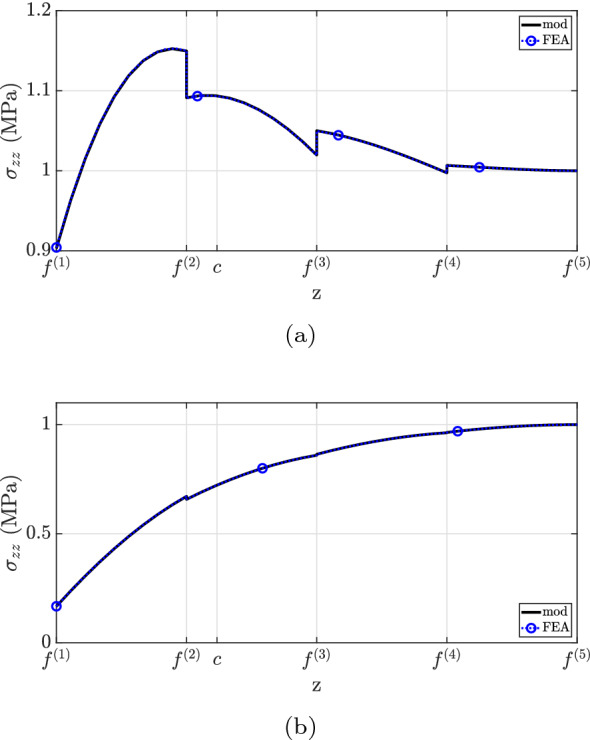
$$\sigma _{zz}$$ for case (3.2): **a** $$x=0.25L$$, **b** $$x=0.75L$$

The transverse direct stress component of the current model and the finite element analysis match the third-order piecewise function depicted in Fig. 22, including the correct magnitude of the pressure load at the horizontal surface. Furthermore, the position of the maximum $\sigma _{zz}$ magnitude is located at the interface between layers $k=1$ and $k=2$ for $x=0.25L$ whereas it is at the upper surface for $x=0.75L$, which represent different positions of maximum shear. Hence, the maximum absolute shear and transverse direct stresses may not occur at the same coordinate.

## Conclusion

An analytical methodology to predict the stress field of untwisted tapered beams comprising multiple isotropic layers has been derived. A state of plane stress with linear and small strains, known internal forces, and perfectly bonded layers, has been assumed. In the scope of laminated tapered beams, new developments include:

1. consideration of beam eccentricity and the first derivative of the longitudinal internal force to define the shear stress profile;
2. recovery of the transverse direct stress component;
3. layerwise body loads and layerwise traction forces;
4. closed-form solutions for the stress field of rectangular cross-sections.

Non-classical terms arise in the transverse stress field for heterogeneous non-prismatic beams due to the arbitrary stiffness distribution through the cross-section. More precisely, the longitudinal external load is typically applied eccentrically to the central axis because the neutral axis position depends on the stiffness distribution through the cross-section. As such, a bending load should be included in the equilibrium relations and considered in the derivation of stress field. Moreover, the term involving the first derivative of the generalized longitudinal stress does not vanish in the shear stress profile of laminated tapered beams, as it would be the case for homogeneous media under generalized external load. In conclusion, the position of maximum shear and transverse direct stresses can not be easily anticipated and may differ from each other. Also, the need to appropriately account for beam eccentricity is required to define the transverse stress field.

The proposed theory is efficient from a computational standpoint as well as processing time being less than finite element analysis, not to mention the analytical formulation does not require mesh convergence studies and the design of each layer as in computer-aided design software. Furthermore, the results suggest excellent agreement with 2D-solid finite element analysis. Practitioners could benefit from the stress field derived in this work to design laminated non-prismatic beams under more complex loading conditions including partial cross-sectional loads, discontinuous loads through the cross-section, pressure and traction forces. Potential further developments involve extending this work to composite laminates, anisotropic media, and functionally graded materials.

## Figures and Tables

**Fig. 23:**
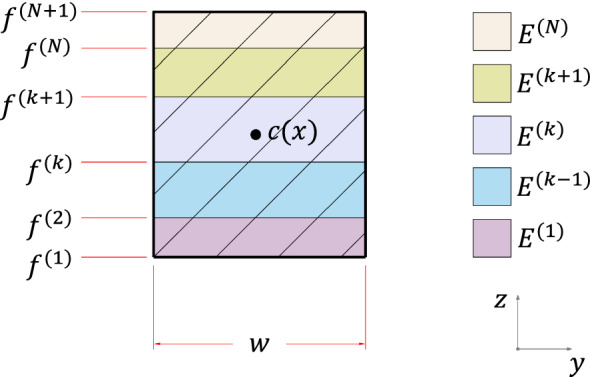
Rectangular cross-section

## Appendix

Closed-form solutions are deduced in this section by defining geometric and stiffness properties for the specific case of rectangular cross-section, illustrated in Fig. 23. An appropriate infinitesimal cross-sectional area can be expressed as d$A=w\mathrm {d}z$, resulting in the following geometric properties

A.1 $$\begin{aligned} \begin{aligned}&\bar{A}=wz \\&\hat{A}^{(k)}=w\left( z-f^{(k)}\right) \\&\phi =\frac{w}{\widetilde{I}}\left( cz-\frac{z^2}{2}\right) \end{aligned} \end{aligned}$$

And the following stiffness properties

A.2 $$\begin{aligned} \begin{aligned}&\widetilde{A} =w\sum _{k=1}^{n}\left( E^{(k)}\hat{f}_k^1\right) \\&\widetilde{S} = \frac{w}{2}\sum _{k=1}^{n}\left( E^{(k)}\hat{f}_k^2\right) \\&\widetilde{I}=w\sum _{k=1}^{n}\left( E^{(k)}\left( c^2\hat{f}_k^1-c\hat{f}_k^2+\frac{1}{3}\hat{f}_k^3\right) \right) \end{aligned} \end{aligned}$$

where $$\hat{f}_k^{n}$$ yields

A.3 $$\begin{aligned} \hat{f}_k^n = \left( f^{(k+1)}\right) ^n-\left( f^{(k)}\right) ^n \end{aligned}$$

The expressions for the Cauchy stress coefficients are given in the following, noting that layer reference (*k*) of the body force, Young’s modulus, and interface functions are omitted to present more concise equations. Expressions for $\eta _i$ are given in the following

A.4 $$\begin{aligned} \begin{aligned}&\eta _{N}=wE\left[ \frac{c'}{2\widetilde{I}}z^2+ \left( \frac{\widetilde{A}'}{\widetilde{A}^2}-\frac{cc'}{\widetilde{I}}\right) z\right] \\&\eta _{(V+m)}=w\frac{E}{\widetilde{I}}\left[ -\frac{z^2}{2} + cz\right] \\&\eta _{M}=-wE\left[ \frac{\widetilde{I}'}{2\widetilde{I}^2}z^2 + \left( \frac{c'}{\widetilde{I}}-\frac{c\widetilde{I}'}{\widetilde{I}^2}\right) z\right] \\&\eta _{N^{'}}=w\left( \frac{F_x}{T_x}-\frac{E}{\widetilde{A}}\right) z \end{aligned} \end{aligned}$$

As Eq. (3.13) suggests, the transverse direct stress requires ${\Delta }\lambda _i$ coefficients to be evaluated at $(x,f^{(k)})$. It is worth mentioning that these coefficients result from differential operations of ${\Delta }\eta _i(x,f^{(k)})$ in Eq. (3.1), which are irrespective of the *z*-coordinate. As such, the values for $\lambda _i$ given in the following are already calculated at $(x,f^{(k)})$

A.5 $$\begin{aligned} \lambda _N&=-E\left\{ \frac{f}{\widetilde{A}^3}\left( 2\left( \widetilde{A}'\right) ^2-\widetilde{A}\widetilde{A}''\right) -f'\frac{\widetilde{A}'}{\widetilde{A}^2} \right. \\&\left. \quad +\frac{f}{2\widetilde{I}}\left[ 4\left( c'\right) ^2+c''(2c-f)\right] \right. \\&\left. \quad +\frac{c'}{\widetilde{I}^2}\left[ f'\widetilde{I}\left( c-f\right) -f\widetilde{I}'(2c-f)\right] \right\} \end{aligned}$$

A.6 $$\begin{aligned} \lambda _{(V+m)}&= E\left\{ \frac{1}{\widetilde{I}}\left[ \left( c - f\right) f' + 2c'f\right] \right. \\&\left. \quad -f\frac{\widetilde{I}'}{\widetilde{I}^2}\left( 2c - f\right) \right\} \end{aligned}$$

A.7 $$\begin{aligned} \lambda _M&=E\left\{ \frac{f}{2\widetilde{I}^3}\left[ 2\left( \widetilde{I}'\right) ^2-\widetilde{I}\widetilde{I}''\right] (f-2c) \right. \\&\left. \quad +\frac{\widetilde{I}'}{\widetilde{I}^2}\left[ (c - f)f' + 2c'f\right] -\frac{1}{\widetilde{I}}(c''f + c'f')\right\} \end{aligned}$$

A.8 $$\begin{aligned} \lambda _{N^{'}}&=E\left[ 2f\frac{\widetilde{A}'}{\widetilde{A}^2} -\frac{f'}{\widetilde{A}} +\frac{c'f}{2\widetilde{I}}(f - 2c)\right] \\&\quad + F_x\left[ \frac{f'}{T_x}-f\frac{T_x'}{T_x^2}\right] \end{aligned}$$

A.9 $$\begin{aligned} \lambda _{V^{'}}&= E\frac{f}{2\widetilde{I}}(2c - f) + \frac{F_z}{T_z} \end{aligned}$$

A.10 $$\begin{aligned} \lambda _{m^{'}}&= E\frac{f}{2\widetilde{I}}(2c - f) \end{aligned}$$

A.11 $$\begin{aligned} \lambda _{N^{''}}&= f\left[ \frac{F_x}{T_x}-\frac{E}{\widetilde{A}}\right] \end{aligned}$$

Finally, expressions for $\zeta _i$ are given in the following

A.12 $$\begin{aligned} \zeta _N&= \frac{E}{6}\left[ 2\frac{c'\widetilde{I}'}{\widetilde{I}^2} - \frac{c''}{\widetilde{I}} \right] z^3 \\&\quad + \frac{E}{2}\left[ 2c'\left( \frac{c'}{\widetilde{I}} - \frac{c\widetilde{I}'}{\widetilde{I}^2}\right) + \frac{cc''}{\widetilde{I}} \right. \\&\left. \quad +\frac{1}{\widetilde{A}^3}\left( 2\left( \widetilde{A}'\right) ^2-\widetilde{A}\widetilde{A}''\right) \right] z^2 \end{aligned}$$

A.13 $$\begin{aligned} \zeta _{(V+m)}&= -E\left[\frac{\widetilde{I}'}{3\widetilde{I}^2}z^3 - \left( \frac{c\widetilde{I}'}{\widetilde{I}^2} - \frac{c'}{\widetilde{I}}\right) z^2\right] \end{aligned}$$

A.14 $$\begin{aligned} \zeta _{M}&= \frac{E}{6\widetilde{I}^3}\left[ \widetilde{I}\widetilde{I}'' - 2\left( \widetilde{I}'\right) ^2\right] z^3 \\&\quad +\frac{E}{2}\left[ \frac{c}{\widetilde{I}^3}\left( 2\left( \widetilde{I}'\right) ^2-\widetilde{I}\widetilde{I}''\right) - \frac{2c'\widetilde{I}'}{\widetilde{I}^2} + \frac{c''}{\widetilde{I}}\right] z^2 \end{aligned}$$

A.15 $$\begin{aligned}&\zeta _{N^{'}}= \left[ E\left( \frac{cc'}{4\widetilde{I}} - \frac{\widetilde{A}'}{\widetilde{A}^2}\right) + F_x\frac{T_x'}{T_x^2}\right] z^2 -\frac{Ec'}{6\widetilde{I}}z^3 \end{aligned}$$

A.16 $$\begin{aligned}&\zeta _{V^{'}} = \zeta _{m^{'}} = E\left[ \frac{1}{6\widetilde{I}}z^3 - \frac{c}{2\widetilde{I}}z^2\right] \end{aligned}$$

A.17 $$\begin{aligned}&\zeta _{N^{''}}=\frac{1}{2}\left[ \frac{E}{\widetilde{A}} - \frac{F_x}{T_x}\right] z^2 \end{aligned}$$

## Alphanumeric characters

*A*(*x*): Cross-sectional area (${\hbox { m}^2}$)
$A^{(k)}(x)$: Cross-sectional area of the *k*th layer (${\hbox { m}^2}$)
$\bar{A}(x)$: Arbitrary cross-sectional area with undefined boundaries (${\hbox { m}^2}$)
$\hat{A}(x)$: Arbitrary cross-sectional area with defined lower boundary (${\hbox { m}^2}$)
$\widetilde{A}(x)$: Generalization of the cross-sectional area multiplied by Young’s modulus (${\hbox { N}}$)
*c*(*x*): *z*-direction distance between the reference system and centroidal position (${\hbox {m}}$)
*E*: Young’s modulus (Pa)
$f^{(k)}(x)$: Functions describing the lower surface of the *k*th layer (${\hbox {m}}$)
$F_i^{(k)}(x,z)$: Body force acting on the *k*th layer (${\hbox {N}/\hbox {m}^3}$)
*h*(*x*): Beam depth ($${\hbox {m}}$$)
$H_f^{(k)}(x)$: Coefficient accounting for traction forces oblique to the coordinate system (${\hbox { m}}$)
$\widetilde{I}(x)$: Generalization of the second moment of area multiplied by Young’s modulus (${\hbox { Nm}^2}$)
*L*: Beam length ($${\hbox { m}}$$)
*m*(*x*): Bending moment due to eccentric axial loading (${\hbox { Nm}/\hbox { m}}$)
*M*(*x*): Bending moment resultant (${\hbox { Nm}}$)
*n*: Number of layers (–)
*N*(*x*): Longitudinal stress resultant (${\hbox { N}}$)
*Oxyz*: Cartesian coordinate system (–)
$\widetilde{S}(x)$: Generalization of the first moment of area multiplied by Young’s modulus (${\hbox { Nm}}$)
$t_i^{(k)}(x)$: Surface force acting on the lower interface of the *k*th layer (${\hbox { N}/\hbox { m}}$)
$T_i(x)$: External load resultant (${\hbox { N}/\hbox { m}}$)
*V*(*x*): Shear stress resultant (${\hbox { N}}$)
*w*: Beam width ($${\hbox { m}}$$)

## Greek characters

${\Gamma }_j(x)$: Terms of the equilibrium of internal forces and their derivatives
${\Delta }(.)$: Discontinuity operator applied to interfaces
$\epsilon$: Discrepancy between results ($\%$)
$\zeta _i^{(k)}(x,z)$: Stiffness-related coefficients necessary for $\sigma _{zz}$
$\eta _i^{(k)}(x,z)$: Stiffness-related coefficients necessary for $\tau _{xz}$
$\kappa _i^{(k)}(x)$: Constants variables originated from undefined integration (${\hbox { Pa}}$)
$\lambda _i^{(k)}(x,z)$: Stiffness-related coefficients necessary for $\sigma _{zz}$
$$\nu$$: Poisson’s ratio (–)
$$\sigma _{ij}$$: Stress tensor ($${\hbox { Pa}}$$)
$\phi (x)$: Ratio between first moment of area in relation to the centroid position and $\widetilde{I}$ (${\hbox { m}/\hbox { N}}$)
${\Omega }^{(k)}$: Domain of the *k*th layer (${-}$)
